# Computed tomography of the spleen in chickens

**DOI:** 10.3389/fvets.2023.1153582

**Published:** 2023-06-01

**Authors:** Yasamin Vali, Michaela Gumpenberger, Cornelia Konicek, Sina Bagheri

**Affiliations:** ^1^Diagnostic Imaging, Department of Companion Animals and Horses, University of Veterinary Medicine, Vienna, Austria; ^2^Department of Companion Animals and Horses, University Clinic for Small Animals, Clinical Unit of Internal Medicine Small Animals, University of Veterinary Medicine, Vienna, Austria; ^3^Clinic for Poultry and Fish Medicine, Department for Farm Animals and Veterinary Public Health, University of Veterinary Medicine, Vienna, Austria

**Keywords:** computed tomography, spleen, poultry, chickens, immune response

## Abstract

The avian spleen is an important immune organ in birds and its size can be used as an index of immune system responses in different conditions. Based on the lack of knowledge in computed tomography of the spleen in chickens, the present study was conducted to assess the inter-and intraobserver reliability in the measurement of the spleen dimensions and attenuation, as well as the feasibility of utilization of these measurements as a predictor of different diseases. For these purposes, the spleens of 47 chickens were included in the study. Two observers measured the dimensions and attenuations of the spleen, which were finally compared with the clinical diagnosis. The results showed an excellent interobserver reliability in the length, width, and height of the spleen (ICC: 0.944, 0.906, and 0.938, retrospectively), and a good interobserver reliability was observed during the evaluation of the average Hounsfield units of the spleen (ICC: 0.818). The intraobserver reliability was excellent in all the measurements (ICC > 0.940). Additionally, no statistical differences were detected in the spleen size and attenuation between the normal and diseased groups. Based on the present results, the computed tomographic measurements of the spleen could not predict the clinical diseases of the chickens; however, the low rates of the inter- and intraobserver variability suggest the reliable utilization of these computed tomographic measurements in routine clinical application and follow-up examinations.

## 1. Introduction

The spleen is an important immune organ in birds as well as other vertebrates, although physiological and anatomical differences exist between avian and mammalian spleens (1, 2). The avian spleen is the main site of lymphocyte differentiation and proliferation, and it plays an active role in hormonal and cell-mediated responses (1). Additionally, the avian spleen is responsible for mounting both innate and adaptive immune responses, which shows the merits of its role in immune regulations (3).

It is common for clinicians and researchers to correlate the size of the spleen with the clinical diseases but there is no published evidence supporting this fact. Also, recently, the spleen size is used as an index of immune system response in infectious diseases or as a biomarker in different immuno-toxicological, ecological, and evolutionary studies, in which the results are not properly translated for clinical use (2, 4–6).

Although the spleen can be evaluated easily through necropsy, diagnostic imaging offers a detailed antemortem evaluation of the spleen’s size, shape, and parenchyma. However, radiography was the pioneer in avian diagnostic imaging (7), nowadays computed tomography (CT) has become much more integrated as a preferred imaging modality in this field (8). CT scans provide clinically useful images by avoiding superimposition of the structures and better contrast resolution (8). Furthermore, the possibility of scanning the coelomic cavity in birds without sedation in a rather short time makes it a meaningful paraclinical examination in avian medicine (8, 9).

To the best of the authors’ knowledge, the sparsely available knowledge of the spleen in avian CT is mainly based on case reports. There is a lack of research that describes the CT features of chicken spleens in normal and diseased patients. Due to this lack of knowledge, this study was conducted to investigate the following aims:

1. To evaluate the size, shape, and attenuation of the parenchyma (HU) of the spleen in normal and diseased chickens;
2. To assess the intra-and interobserver agreement on the objective evaluation of the spleen in plain CT;
3. To present reliable indices for evaluation of the chicken spleen size.

We hypothesized that, first, the spleen size, attenuation (HU), and suggested indices differ in normal and diseased chickens; second, more specific differences may exist between different pathological conditions; and, finally, there is an excellent interobserver agreement in the objective evaluation of this organ.

## 2. Materials and methods

The study was a retrospective design, so no institutional animal care and use approvals were requested officially and written informed consent was obtained from the owners for the participation of their animals potentially in retrospective studies at the time of admission in the hospital.

The archives of the diagnostic imaging section were investigated retrospectively between 2014 and 2020 for all chickens that underwent CT in the Department of Small Animal and Horses, University of Veterinary Medicine of Vienna. The CT scans were performed using a SOMATOM® Emotion 16-slice helical CT scanner (Siemens Healthcare, Erlangen, Germany). Consistent scan parameters used included 130 kV tube potential, 80–100 mAs, 0.6 mm slice width, and helical scan mode with a collimator pitch of 0.8. For reconstruction, a soft tissue kernel as well as a sharp bony kernel were used. Chickens were included in the study if the entire coelomic cavity was included in the field of view (FOV) and the quality of the images was recorded “diagnostic” by a third-year resident of ECVDI (YV). The included chickens were categorized based on the final diagnosis and laboratory results recorded in their medical history into five main groups: 1. normal or trauma or orthopedic patients, 2. infection, 3. inflammatory disorders with unknown origin, 4. neoplasia, 5. egg binding, and 6. egg peritonitis. The “infection category” was categorized into bacterial, viral, fungal, and parasitic infection subcategories.

The gender of the included cases was extracted from the medical record for further statistical analysis. All the images were reviewed using the soft tissue kernel reconstruction with the assessed window width (WW) and window level (WL), depending on the preference of the observers using the Osirix_Lite® software (version 12.x, Pixmeo SARL, Switzerland). The cases were excluded during the evaluation if the spleen was displaced or effaced due to the crowded celomic cavity (several large follicles or abnormal mass). The observers were not aware of signalments and the final diagnosis of the cases.

## 3. Objective evaluation

**Figure 1:**
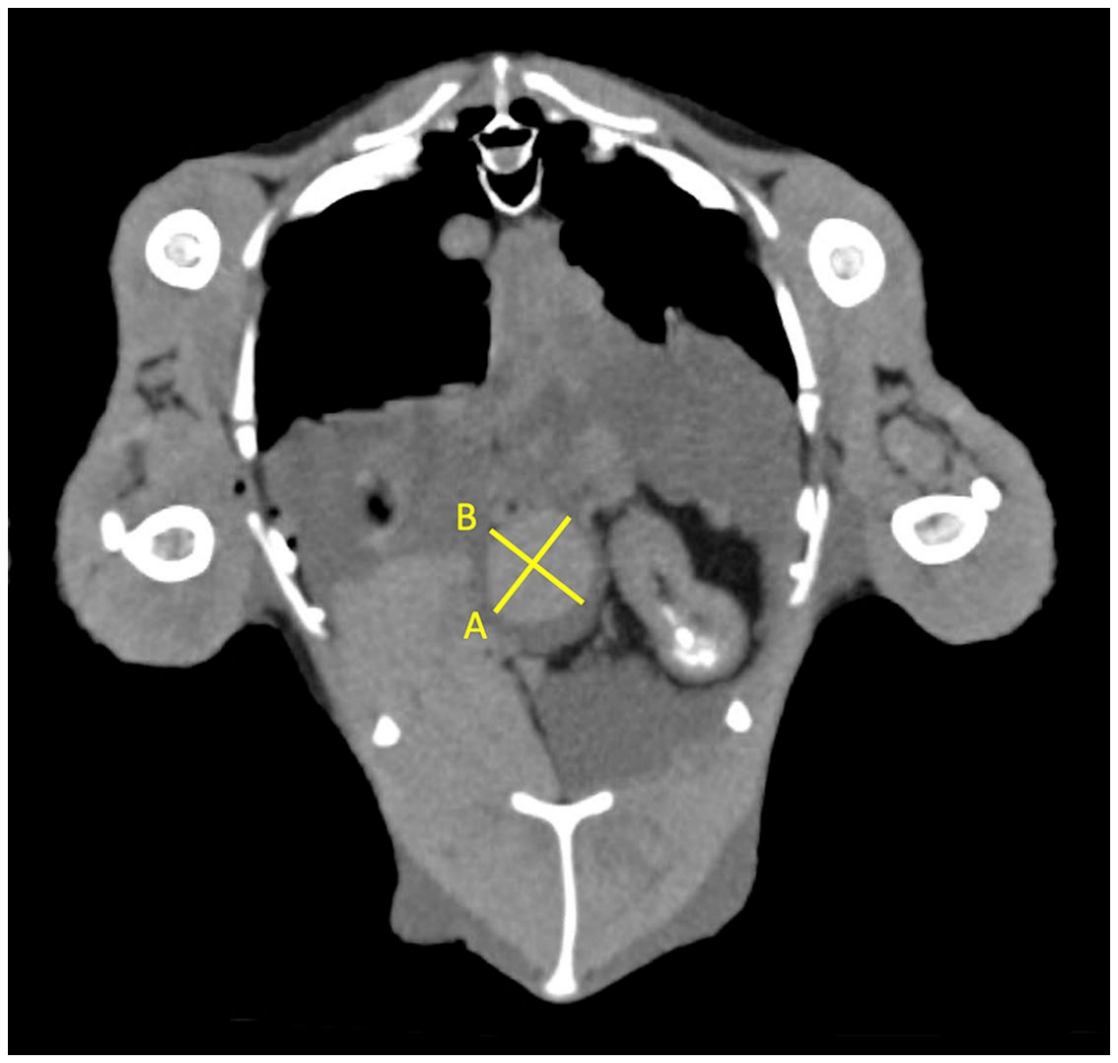
Transverse plane of the coelomic cavity at the level of the spleen. The width measured as the widest dimension of the spleen **(A)** and the height measured as the dimension perpendicular to the previously measured width **(B)**.

**Figure 2:**
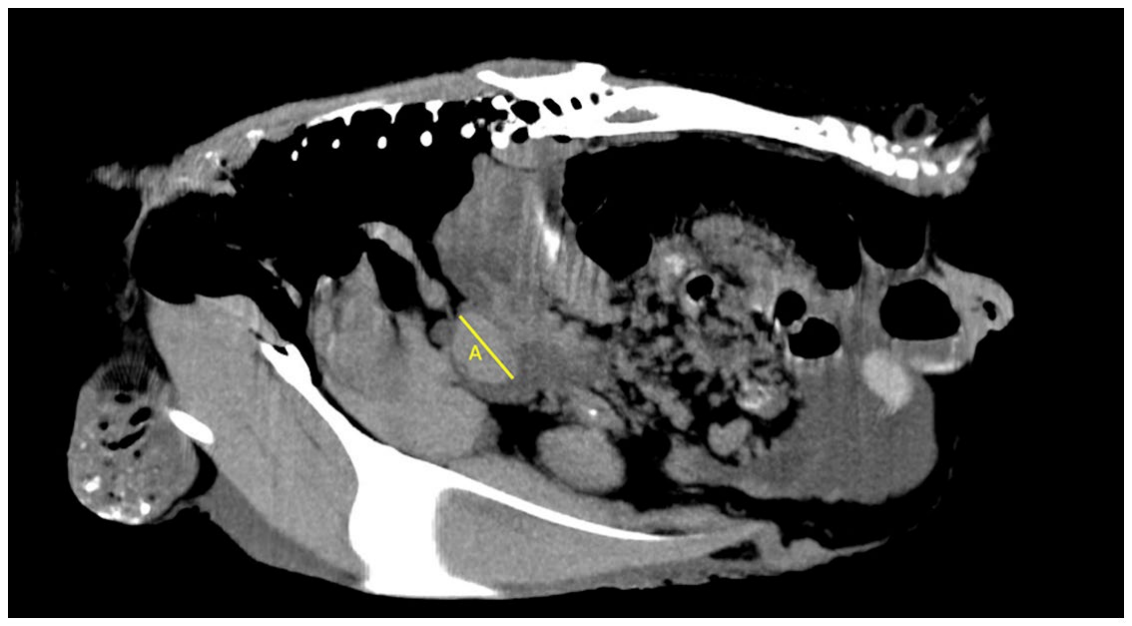
Sagittal plane of the coelomic cavity at the level of the spleen. Length of the spleen (A).

**Figure 3:**
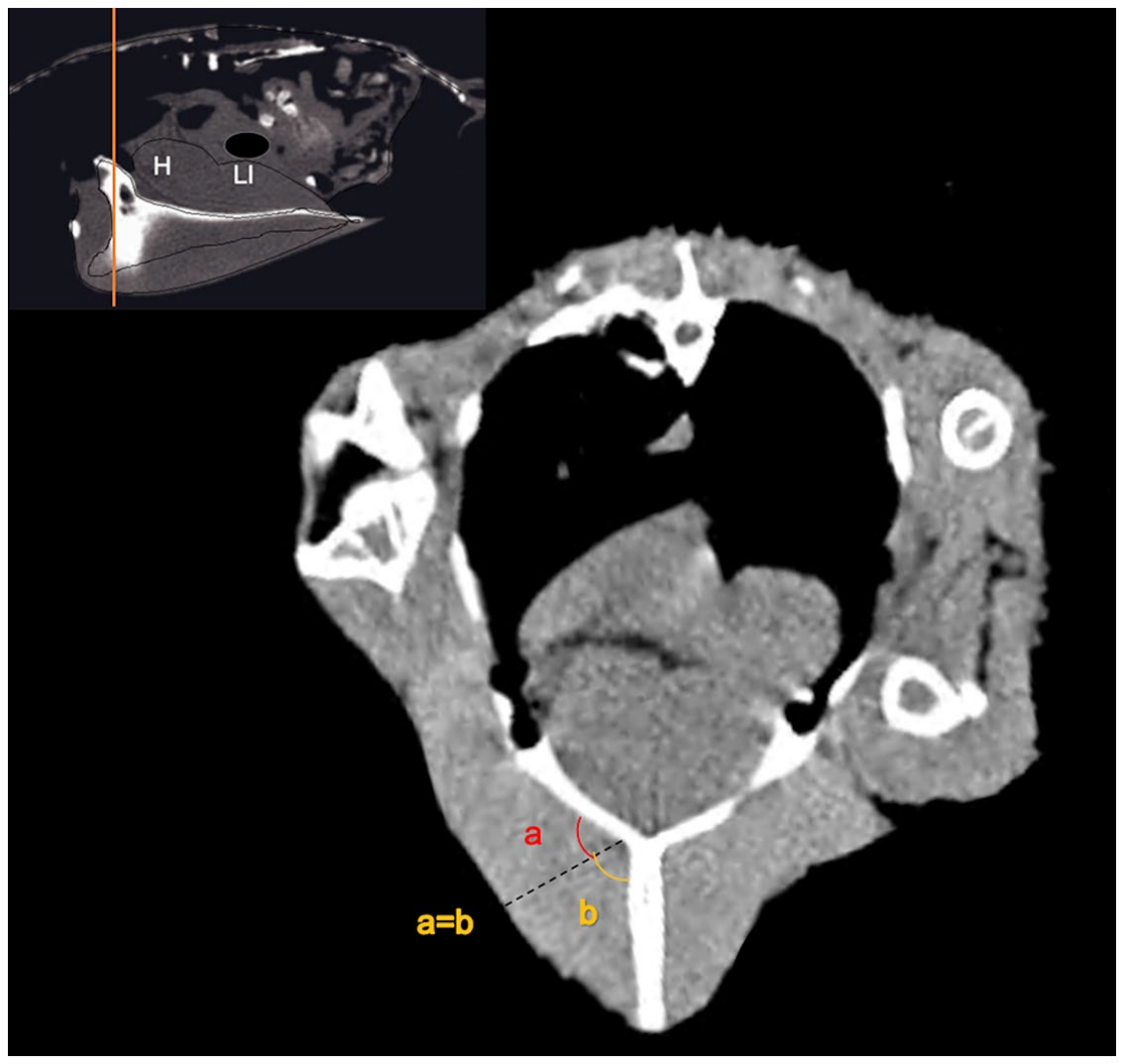
The thickness of the pectoral muscle was measured on both sides using the transverse plane just caudal to the cranial curvature of the keel (note the guide picture in the upper left corner). The caliper was placed from the junction of the sternal keel to the surface bisecting the angle (a = b).

In total, two veterinary radiologists evaluated the included cases: a third-year resident of ECVDI with experience in avian diagnostic imaging (YV) and an assistant professor specialized in avian and reptile diagnostic imaging (MG). The same guidelines were used by these two radiologists for the objective evaluation (Figures 1–3). The measurements were repeated by the first observer (YV) after 8–12 months. The images were reconstructed in multiplanar reconstruction (MPR) based on the anatomical axis of the celomic cavity with the sagittal and coronal plane oriented parallel to the spine. The width and height of the smoothly outlined, oval-shaped spleen were measured in a transverse plane. The width was measured as the widest dimension of the spleen (not necessarily parallel to the horizontal MPR axis) (Figure 1). Then, the height was measured as the dimension perpendicular to the previously measured width (Figure 1). The length was measured as the longest dimension of the spleen in the sagittal plane (not necessarily parallel to the horizontal MPR axis) (Figure 2).

The attenuation of the spleen (Hounsfield Unit, HU) was measured by drawing a circle in the center of the spleen (region of interest, ROI) in the transverse plane. The ROI was placed a minimum of 1–2 mm far from the borders. Then, the minimum of HU (MinHU), the maximum of HU (MaxHU), the average of HU (AvHU), and the standard deviation of HU (SDHU) were recorded.

The thickness of the pectoral muscle (MTH) was measured on both sides using the transverse plane just caudal to the cranial curvature of the keel. The caliper was placed from the junction of the sternal keel to the surface bisecting the angle (Figure 3). The attenuation of the muscles was measured on both sides by an ROI. The ROI was placed in the center minimum of 1–2 mm far from the borders to record MinHU, MaxHU, AvHU, and SDHU, similar to the spleen.

The pectoral muscle’s thickness was used as an indicator of body condition. Furthermore, it served as a rating tool together with the spleen size to create quantitative indices and ratios.

Consequently, three ratios were suggested by dividing the spleen width, length, and height by the pectoral muscle’s thickness (WS/MTH, LS/MTH, and HS/MTH, respectively) and included in the measurements for further statistical analysis.

## 4. Subjective evaluation

As the body condition score (BCS) was not recorded in the medical records of the cases, thus CT images were used for subjective scoring. A poultry specialist, with a doctorate in avian and poultry medicine and PhD candidate in poultry medicine (SB), recorded the body condition score of each patient by subjective evaluation of the pectoral muscle in the transverse plane based on the keel scoring guideline (10).

For the subjective evaluation of the spleen’s shape, the dorsal plane was used, while the MPR plane was adjusted on the length of the spleen. Then, the area of the spleen was measured using the Adobe Photoshop® CS5 (Extended Middle Eastern Version 12.0× 32, Adobe Inc.). Each spleen was colored with 10% opacity of black and layered on another to present the morphological variation, the common area, and the shape of the spleens.

## 5. Statistical evaluation

The data were collected and analyzed using SPSS 10.0 for Windows (SPSS Inc., Chicago, IL, United States) by a third-year resident of ECVDI under the supervision and guidance of a statistician, PhD in biostatistics (EL). An agreement between intra-and interobserver measurements was assessed by the intraclass correlation coefficients (ICCs) using a two-way mixed-effects model. ICC values less than 0.5, between 0.5 and 0.75, between 0.75 and 0.9, and greater than 0.90 were interpreted as poor, moderate, good, and excellent reliability, respectively (11). *P*-values below 0.05 were considered statistically significant. The normality of the data was assessed by the Shapiro–Wilk Test. The results of measurements (spleen dimensions and attenuation) and suggested indices were compared with the recorded final diagnosis by the Kruskal–Wallis test. The correlation of the spleen dimensions and HU in relation to muscles’ measurements was assessed by the Spearman rank-order correlation coefficient. Principal component analysis (PCA) was used to illustrate the relationship among the three dimensions of the spleen, the abovementioned six different disease categories, two different groups (normal/trauma and the rest of the disease groups), gender, and BCS. PCA was performed by R software (R Core Team (2019), version 3.6.1. R: A language and environment for statistical computing. R Foundation for Statistical Computing, Vienna, Austria. URL http://www.R-project.org/).

## 6. Results

**Figure 4:**
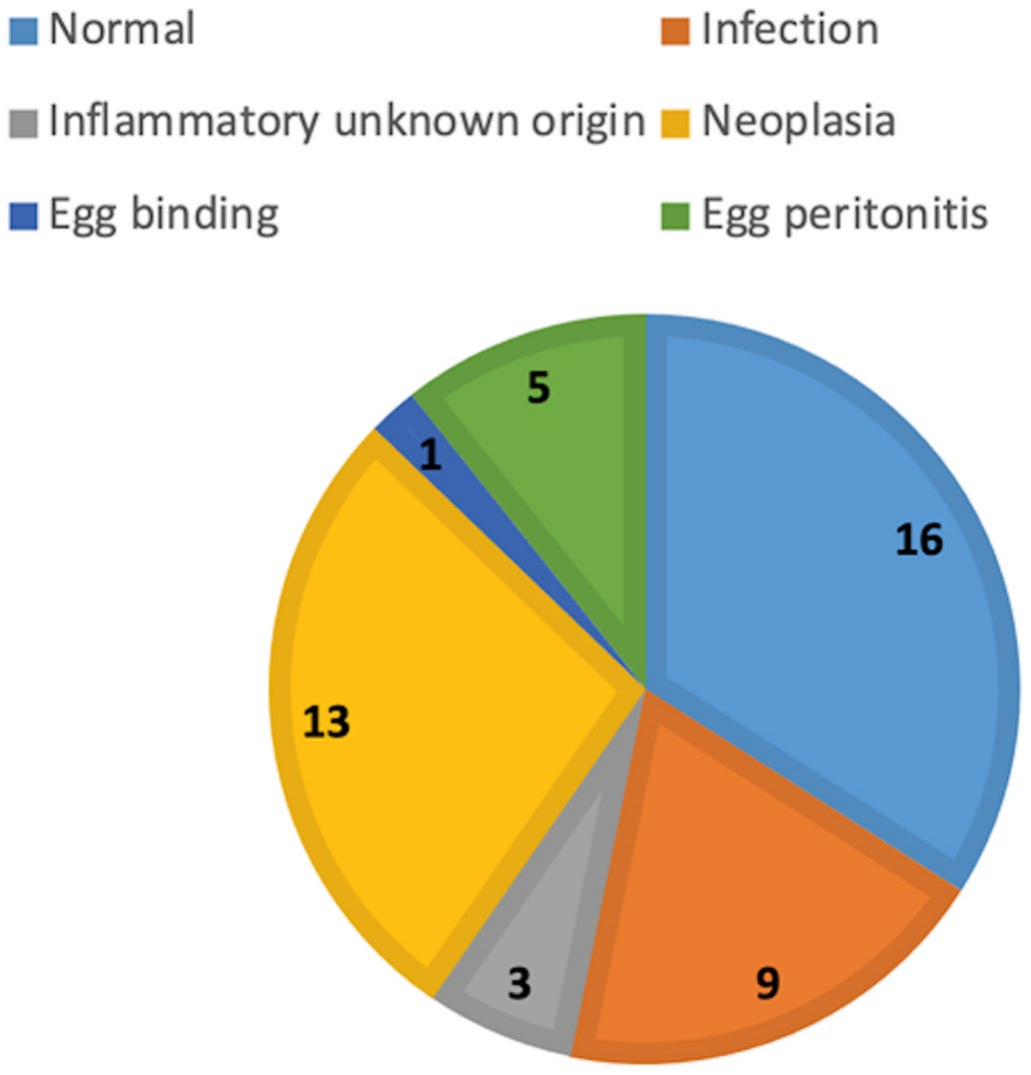
The number of chickens in each group.

**Figure 5:**
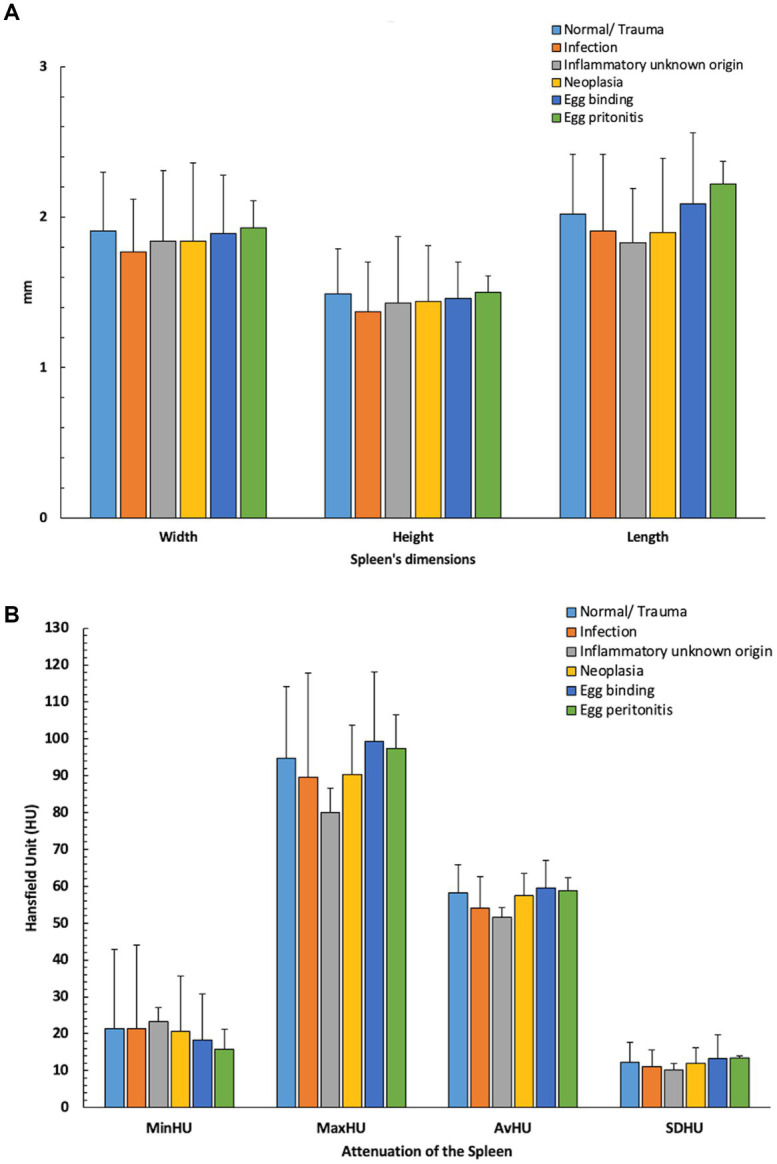
Means ± SD of the objective measurements of the spleen [**(A)**: dimensions and **(B)**: attenuation] in different groups. Minimum (Min); Hounsfield Unit (HU); Average (Av); Standard Deviation (SD).

Overall, 50 chickens were included during the first archive screening and three of them were excluded during the evaluation due to crowded coelomic cavities. Finally, objective and subjective evaluations were carried out in 47 chickens. The number of chickens in different groups was unequal (Figure 4). Means ± SD of the objective measurements of the spleen (dimensions and attenuation) was calculated for further statistical evaluation (Figure 5).

An excellent interobserver reliability was found in measuring the length (ICC 0.944, 0.858–0.974, *p*-value<0.001), width, and height of the spleen (ICC 0.038, 0.791–0.974, p-value<0.001). The average measure ICC was calculated as 0.940 and 0.958 with a 95% confidence interval from 0.861 to 0.971 and 0.921 to 0.977 for the left and the right pectoral muscles, respectively, for the abovementioned measurements (*p* < 0.001). Additionally, a good interobserver reliability was observed during the evaluation of the AvHU of the spleen. The average ICC was 0.818 with a 95% confidence interval of 0.673–0.898 (*p* < 0.001). The intraobserver reliability was excellent in all the measurements (Table 1).

**Table 1:** Intraobserver reliability.

Measurement	Average ICC	Range
Height of spleen	0.983	0.939–0.993
Width of spleen	0.961	0.929–0.978
Length of spleen	0.985	0.961–0.993
MaxHU of spleen	0.901	0.821–0.945
MinHU of spleen	0.960	0.929–0.978
AvHU of spleen	0.950	0.910–0.972
SDHU of spleen	0.958	0.925–0.977

Inter Class Correlation (ICC) with 95% confident interval and *p-*value<0.00 for all the measurements. Minimum Hounsfield Unit (MinHU), maximum Hounsfield Unit (MaxHU), average Hounsfield Unit (AvHU), standard deviation of Hounsfield Unit (SDHU).

Based on the good agreement between the observers, the measurements by the first observer were used for further statistical analysis. The largest mean was reported for the spleen’s width in the viral infection group (Mean = 1.98 ± 0.01 cm, *n* = 3), for the spleen’s height in the normal/traumatic group (Mean = 1.49 ± 0.3 cm, *n* = 16), and for the length of the spleen in the egg peritonitis group (Mean = 2.08 ± 0.47 cm, *n* = 5). The highest MinHU of the spleen was calculated in the parasitic infection group (Mean = 27.71 ± 9.7 HU, *n* = 7), the highest MaxHU and SDHU were detected in the viral infection group (Mean = 108 ± 46.36 HU, *n* = 3; and Mean = 14.74 ± 6.7 HU, *n* = 3, respectively), the highest AvHU was calculated in the egg peritonitis group (Mean = 59.50 ± 7.05, *n* = 5) (Table 2). Moreover, the Kruskal–Wallis H-test showed no statistically significant difference in the measured parameters between the different groups and subcategories of the diseases.

**Table 2:** Spearman’s rank-order correlation between the width, height, and length of the spleen.

	Width	Height	Length
Width	–	0.80^**^	0.60^**^
Height	0.80^**^	–	0.730^**^
Length	0.60^**^	0.730^**^	–

^**^p-value < 0.001.

The Kruskal–Wallis H-test showed a statistically significant difference in the thickness of muscles between males and females, χ2(2) = 6.151, *p* = 0.013, with a mean rank thickness of muscle of 21.93 for females and 35.86 for males. Thus, subsequently the suggested ratios in the present study containing the thickness of the muscles showed a significant difference between males and females. No statistically significant differences were detected in other parameters between females and males.

The Kruskal–Wallis *H*-test showed that there was a statistically significant difference in the suggested ratios (WS/ MTH, HW/ MTH, and LW/ MTH). WS/ MTH was different between different BCSs, χ2(2) = 11.131, *p* = 0.011, with a mean rank ratio WS/MTH of 30.32 for BCS 2, 16.31 for BCS 3, 18.33 for BCS 4, and 19.60 for BCS 5. HS/MTH was also different between different BCSs, χ2(2) = 8.952, *p* = 0.030, with a mean rank ratio HS/MTH of 29.68 for BCS 2, 18 for BCS 3, 17,67 for BCS 4, and 17.40 for BCS 5. Additionally, a significant difference was detected in ratio LS /MTH between different BCSs, χ2(2) = 13.784, *p* = 0.003, with a mean rank ratio LS/MTH of 30.86 for BCS 2, 18.69 for BCS 3, 11 for BCS 4 and 14 for BCS 5. No statistically significant differences were detected in other parameters of different BCSs.

A positive correlation exists between the width, height, and length of the spleen (Figure 4). Furthermore, Spearman’s rank-order correlation showed a positive correlation only between the width of the spleen and the Max HU of the spleen (*r* = 0.339, *n* = 14, value of *p* = 0.02).

**Figure 6:**
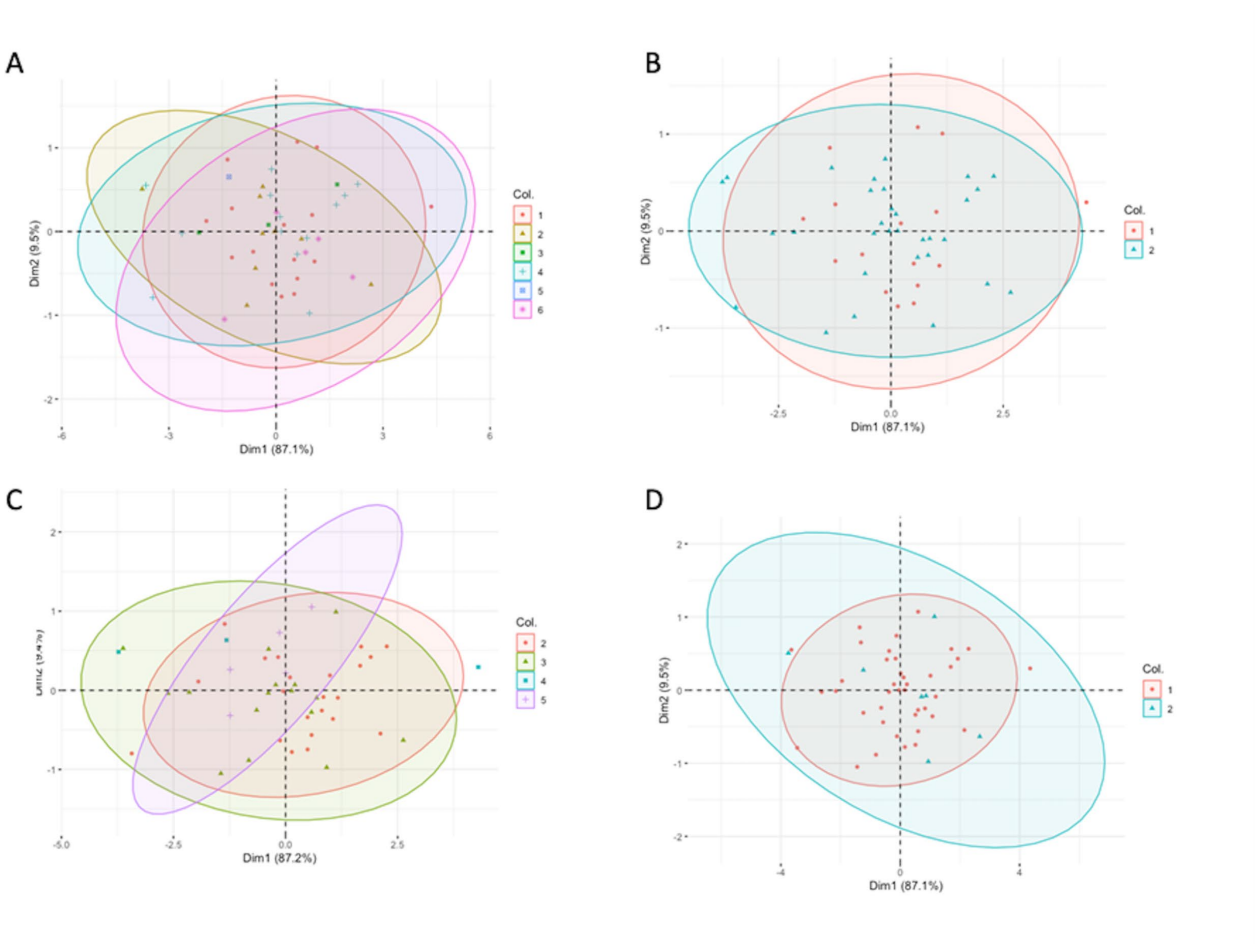
Principal component analysis showing the relationship of the three dimensions of the spleen coloring by **(A)** six different groups, **(B)** two different groups: normal and diseased groups, **(C)** different body condition scores, and **(D)** different gender. **(A)** 1. Normal/ trauma, 2. infection, 3. inflammatory with unknown origin, 4. neoplasia, 5. egg binding and 6. egg peritonitis. **(B)** 1. Normal/ trauma, 2. diseased group (infection, inflammatory with unknown origin, neoplasia, egg binding, egg peritonitis as a same group). **(C)** The numbers present the different body condition scores **(D)** 1. Male, 2. Female.

The PCA showed a large overlap of the spleen dimensions in different groups of diseases, BCS, and genders (Figure 6). Unfortunately, no specific pattern was found in the spleen’s dimensions related to specific diseases, BCS, and different genders.

**Figure 7:**
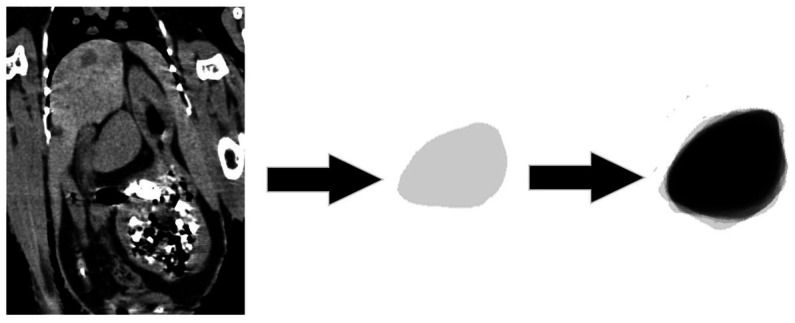
Morphologic variation of the spleen. The spleen of each chicken is colored with a 10% opacity of black. The darker region shows a common shape of the spleen in all the patients.

Finally, Figure 7 presents the morphologic variation of the spleens, in which the darker region shows a common area in all the cases. The general shape is, however, blunted ellipsoid.

## 7. Discussion

The present study revealed no statistical differences in the spleen size, attenuation, and suggested indices between the normal and diseased groups. The results showed an excellent interobserver reliability in measuring the thickness of the pectoral muscles as well as the length, width, and height of the spleen, while a good interobserver reliability was observed during the evaluation of the AvHU of the spleen. The intraobserver reliability was excellent in all the other measurements.

The role of the spleen is fully understood in mammals, and the probable causes of splenomegaly are well described (10); however, the interspecies differences should be taken into account. The avian spleen plays no role in erythrocyte storage and has limited activity in erythropoiesis, so the spleen is mainly considered as an immune organ with a direct relation between the spleen mass and immunocompetence (4). Moreover, as the spleen is mainly composed of water, so the terms “spleen volume,” “spleen weight,” and “spleen size” can be used interchangeably (12). Accordingly, a part of the hypotheses of the present study was proposed based on the main immunological role of the avian spleen and to prove the potential of using the spleen size in computed tomography to diagnose and differentiate the different infection diseases from normal spleen and neoplastic infiltration of the spleen, which unfortunately failed.

Similar to other species, the assessment of the avian splenic size is mainly subjective (10). Because there is no specific threshold to diagnose a pathologically enlarged spleen and no agreement on what constitutes a “normal” spleen in birds, a subjectively prominent spleen is often reported as “splenomegaly” by radiologists. In the present study, no significant differences were reported between the spleen size in the normal/trauma group and the diseased groups, and, hence, we failed to present a range for the normal spleen due to a large overlap in the spleen size between the groups.

Furthermore, the spleen size has a high variability among individuals, it can be also affected by several factors and conditions (13). The splenic size can increase not only during inflammation and infections, but enlargement was also reported previously in older birds (13), broiler breeds, in males (2, 14), and by increasing growth rate (13, 15). On the contrary, stress levels (16) and sex hormones in high breeding activity suppress the immune system (17) and consequently decrease the spleen size. Moreover, these factors can act interrelated. Therefore, we should consider that they may affect the spleen size independently. The chickens in the present study were referral chickens in 6 years with different breeds, husbandry conditions, and diets, thus the effects of the abovementioned factors are not included in the present study and may partly explain the lack of differences between the normal and diseased groups. On the other hand, this reflects daily reality.

Basically, there are many controversial theories to explain the size of the spleen. As the factors that reduce the spleen size have a direct effect on the suppression of the immune response (18), it might then lead to higher bacterial, viral, or parasitic infections, which will increase the spleen size. Therefore, it is difficult to establish a clear cause-and-effect relationship in cases where there is a change in spleen size along with an infectious disease. It is important to take into account that compensatory factors that can decrease or increase the spleen size may mask significant changes in size. In addition, no statistical differences were observed between the parasitic infection group and the other groups, although a direct relationship between the splenomegaly and parasite load in birds was reported previously (2, 19). The possible reason to explain this finding is that the included birds in the present study were all backyard chickens and the final clinical diagnosis was used for further statistical analysis, and it is not possible to present with certainty that the patient was not involved with the other concurrent infections subclinically. Hence, only one factor should not be considered as a significant reason for splenomegaly. The further investigation of the effect of different diseases and pathological conditions needs designing a study on the specific pathogen-free (SPF) chickens and challenging them with an individual specific pathogen.

However, CT is a diagnostic imaging modality with an excellent reproducibility for objective evaluation of nodules and small structures especially larger than 10 mm, while measurement of small irregular structures stays challenging (20, 21). The current study showed an excellent and good reproducibility for measuring the spleen dimensions using the same guideline. This result may represent the merits of using the constant guideline for the objective evaluation of geometrically asymmetrically shaped structures such as the avian spleen.

Smith (2004) emphasized on the necessity of presenting valid indices to evaluate the spleen size (1). In the present study, we used the pectoral muscles’ thickness as representative of the body score of the patient to adopt the spleen size. Unfortunately, no significant differences were detected between the normal and different diseased groups in the suggested ratios.

In the latest part of the subjective evaluation of the spleen by overlying the shapes of the spleens in all the birds, the common shape was almost the shape of a pistachio nut, which also was presented previously by John in 1994 (2). Additionally, the presented correlation between the dimensions of the spleen (Table 2) shows that the spleen enlarged or shrank in all the dimensions relatively.

The lack of research that described the CT features of the chicken spleens in normal and diseased patients limits a proper discussion and comparison of the findings. An additional limitation of this study is the absence of any pathological examination (as the chicken survived), aside from the abovementioned intrinsic limitation due to the retrospective inclusion of the referral patients and therefore unequal sample size in the different diseased groups. Accordingly, the spleens were not removed and evaluated grossly for true size, nor were the spleen tested histopathologically. Thus, it should be considered that more than one disease process may apply to each bird.

In conclusion, measuring the spleen dimensions and attenuation has high reproducibility using the same guideline, thus the authors suggest their guideline for routine clinical application. Based on the present results, the computed tomographic measurements of the spleen could not predict the clinical disease of the chickens; however, this result may be affected by the limitations of the retrospective nature of this study. Hence, further evaluations are recommended to evaluate the effect of the different diseases on spleen presentation.

## Data availability statement

The raw data supporting the conclusion of this article will be made available by the authors, without undue reservation.

## Ethics statement

Ethical review and approval was not required for the animal study because the study was a retrospective design, so no institutional animal care and use approvals were requested officially and a written informed consent was obtained from the owners for the participation of their animals potentially in retrospective studies at the time of admission in the hospital. Written informed consent was obtained from the owners for the participation of their animals in this study.

## Author contributions

YV and SB: conception and design. YV, MG, CK, and SB: acquisition of data, revising article for intellectual content, and agreement to be accountable for all aspects of the study in ensuring that questions related to the accuracy or integrity of any part of the study are appropriately investigated and resolved. YV and SB: analysis and interpretation of data. YV and SB: drafting the article. All authors contributed to the manuscript and approved the submitted version.

## Funding

The Article Processing Charges (APC) for the current manuscript was paid from the resources of the Open Access Fund of the University of Veterinary Medicine Vienna (Vetmeduni).

## Conflict of interest

The authors declare that the research was conducted in the absence of any commercial or financial relationships that could be construed as a potential conflict of interest.

## Publisher’s note

All claims expressed in this article are solely those of the authors and do not necessarily represent those of their affiliated organizations, or those of the publisher, the editors and the reviewers. Any product that may be evaluated in this article, or claim that may be made by its manufacturer, is not guaranteed or endorsed by the publisher.
